# Use of a novel coaxial guide needle-wire (GNW) combination system for computed tomography guided radiofrequency tumor ablation

**DOI:** 10.1186/1477-7819-9-127

**Published:** 2011-10-13

**Authors:** Hiroyuki Tokue, Yoshito Tsushima, Hiroshi Ishizaka, Azusa Nakazawa

**Affiliations:** 1 Department of Diagnostic and Interventional Radiology, Gunma University Hospital, Maebashi, Gunma, Japan; 2 Department of Radiology, Maebashi Red Cross Hospital, Maebashi, Gunma, Japan

## Abstract

We developed a novel coaxial system using a fine guide needle wire (GNW) to safely and easily place the radiofrequency needle under CT-guidance. The GNW consists of a fine needle (diameter, 21-gauge; length, 150 mm) and a wire (0.018 inch, 250 mm). An exclusive radiofrequency cannula (14-gauge; 160 mm) was also used. This system was used for the treatment of six hepatocellular carcinomas in six patients. All lesions were located deeper than 10 cm from the needle entry site. This system was useful in performing CT-guided RF ablation for deeply or precariously located liver lesions particularly in patients who are unable to hold their breath.

## Background

Computed tomography (CT) guided percutaneous radiofrequency (RF) tumor ablation plays an important role in the treatment of patients when lesions cannot be detected by ultrasonography. However, with CT guidance, accurate RF electrode targeting of relatively small gauge for deeply situated lesions is occasionally difficult. In particular, the weight of the RF needle makes step-by-step CT-guided angular manipulations complicated. In RF ablation, a coaxial needle system way facilitates pre-procedural planning and lesion mapping [1]. In addition, it allows pre-treatment biopsy through the same percutaneous channel. However, it may be dangerous or difficult to puncture a lesion using a thick coaxial needle by try to trial, if the lesion is located near a vessel or the pleura [2,3]. On this account, we supposed to believe that it may to will be safer to use two-step coaxial technique using a fine guide-needle. But two-step coaxial technique requires long guide-needle if the lesion is deeply and precariously located, and a long guide-needle is intricate to apply within the narrow CT gantry space. We developed a novel coaxial system using a fine guide needle wire (GNW) to safely and easily place the RF needle under CT-guidance.

## Materials and methods

This system was used for six lesions in six patients (two men and four women; mean age, 70 years; range, 62-77) with hepatocellular carcinoma (HCC). All lesions were located deeper than 10 cm from the needle entry site (mean length, 12 cm; range, 10-15 cm). Hemocoagulation parameters in all patients were within normal limits. In addition, we used GNW for trans-hepatic drainage in deeply located abscess in one patient.

Before the therapy, an informed consent was obtained from all patients after detailed information and explanations concerning the effect and potential risks.

A conventional CT imager (X-force/SH, Toshiba Medical, Tokyo, Japan) was used for image guidance. The needle entry site and the degree of inclination needed to direct the needle into the lesion was determined by initial localization scans obtained during a metallic marker positioned on the patients' body surface.

**Figure 1:**
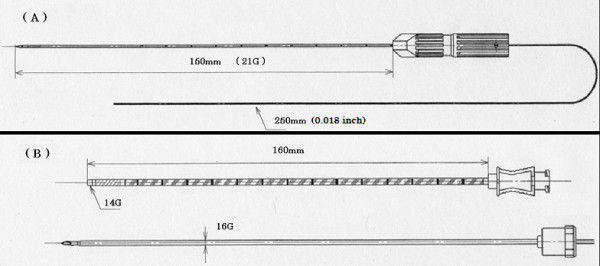
**The novel fine guide needle wire (GNW) (A) and coaxial system (B)**.

**Figure 2:**
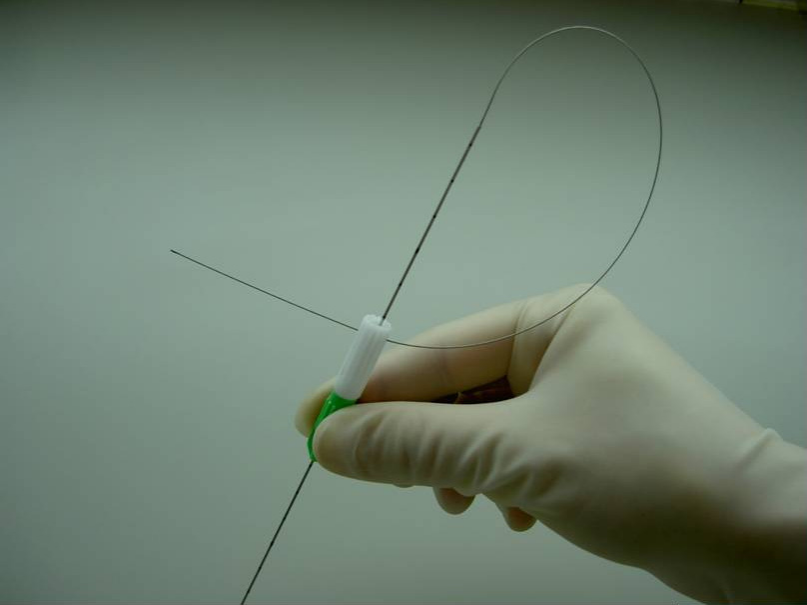
**Picture of the novel fine guide needle wire (GNW)**. The needle and the wire are welded. The guide needle wire is attached a removable hub. The wire can bend freely.

We got the cooperation of the company (Hakko. Co. LTD, Nagano, Japan) and GNW was made by the company. GNW consists of a fine needle (21-gauge) and a wire (0.018 inch) of stainless steel (Figure 1). The needle part is tubular type, and the tip of the needle side is closed. The wire is fitted in the needle, and it can not fall out. The length of the needle part is 150 mm, scaled every 10 mm, and the length of the wire part is 250 mm. The wire can bend freely. GNW is attached a removable hub, and the wire can put through the hole of the hub at the time of insertion. (Figure 2) The curve of GNW during insertion can also be enhanced slightly by gently angling the tip with the fingers before insertion. GNW was advanced until its tip penetrated the target. When GNW was used in conjunction with CT guidance, GNW was advanced in a stepwise manner with quick applications of conventional helical 5-mm imaging or interrupted CT-fluoroscopic display to check the position of GNW tip. The coaxial needle (an exclusive RF cannula (14-gauge; 160 mm)) was then advanced along GNW. After the procedures with CT-guidance and RF ablation therapy were completed, immediate follow-up CT was performed.

## Results

**Figure 3:**
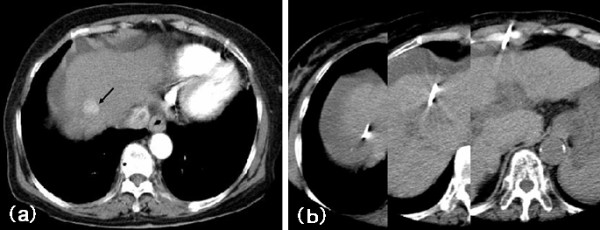
**A 76-year-old woman**. Arterial-phase CT shows cirrhotic liver with a highly enhanced HCC in the right lobe (arrow) and a small amount of ascites (Fig. 3a). The needle entry site was decided to avoid through acites and right lung. GNW was accessed to HCC (Fig. 3b). The lesion was located 11 cm from the needle entry site.

GNW was easily accessed to the lesions and the outer needle could be advanced along the guide needle-wire within the CT gantry in all cases without difficulty (Figure 3). No complications associated with the use of the coaxial system were observed. Six months later, follow-up was carried out with the enhanced CT. There were no local recurrences in all patients.

**Figure 4:**
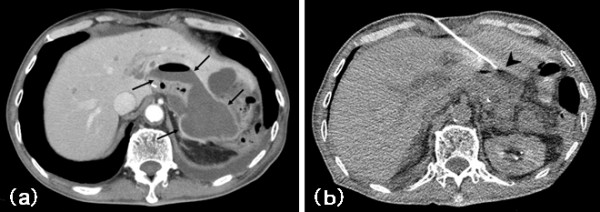
**A 76-year-old man was operated for the gastric-cancer 7 days ago**. Enhanced CT shows abscess (arrows) in the operated area (Fig. 4a). GNW was accessed to abscess (arrowhead) (Fig. 4b). We used this accessed route for drainage. The lesion was located 9 cm from the needle entry site.

In addition, GNW were able to be modified for trans-hepatic drainage (Figure 4).

## Discussion

Two-step coaxial technique is useful by for deeply situated lesions. However, this technique requires a long guide-needle (length of exceeding double of depth of lesion) and is intricate to apply within a CT gantry space.

GNW combination used in our study is easy to manipulate within a gantry space because of a short needle segment. Unlike the conventional two-step procedure, in which one first uses a guide-wire then exchanging to the outer needle, the outer needle can be steadily advanced along GNW in our new method. In our experience using the conventional two step method, the flexible wire may occasionally be pushed back to the liver surface due to resistance when intending to advance the outer needle along the wire.

Using GNW has some advantages. GNW is thinner and longer than those already available in the market today (for example, a coaxial LeVeen needle electrode; 14 gauge, 2-to 4- cm array diameter) [1]. We support that its use is easier and safer to use than those already existing, when multiple needle manipulations are required [4]. The light GNW also facilitated step-by-step CT-guided angular manipulations, unlike heavy RF electrodes, which are unstable during hands-free use unless deeply inserted. GNW can be applied to other interventions, such as biopsy and trans-hepatic drainage, in cases conventionally contraindicated because of high risk [5]. The disadvantages of GNW are that the sample is very small and no statistic analysis of any kind (time, dose or number of CT images, complications etc) in comparison with results obtained with other devices [6].

GNW system enables us to perform safely and easily CT-guided works for deeply and precariously located lesions even in patients who are unable to hold their breath. And we suggest that GNW can be modified for trans-hepatic drainage.

## Conclusion

This new system is useful in performing CT-guided RF ablation for deeply or precariously located liver lesions, particularly in patients who are unable to hold their breath. We, therefore propose this system for safe and efficient treatment on difficult to access lesions.

## Competing interests

The authors declare that they have no competing interests.

## Authors' contributions

HT reviewed relevant literature and drafted the manuscript. All authors provided clinical expertise and participated in drafting the manuscript. And all authors read and approved the final manuscript.
